# Classical Swine Fever Virus Structural Glycoprotein E2 Interacts with Host Protein ACADM during the Virus Infectious Cycle

**DOI:** 10.3390/v15051036

**Published:** 2023-04-23

**Authors:** Elizabeth Vuono, Elizabeth Ramirez-Medina, Ediane Silva, Keith Berggren, Ayushi Rai, Nallely Espinoza, Douglas P. Gladue, Manuel V. Borca

**Affiliations:** 1 Plum Island Animal Disease Center, Agricultural Research Service, United States Department of Agriculture, Greenport, NY 11944, USA; 2 Department of Pathobiology and Population Medicine, Mississippi State University, Starkville, MS 39762, USA; 3 Oak Ridge Institute for Science and Education (ORISE), Oak Ridge, TN 37830, USA

**Keywords:** CSFV, classical swine fever (CSF), E2 glycoprotein, virus virulence

## Abstract

The E2 glycoprotein is one of the four structural proteins of the classical swine fever virus (CSFV) particle. E2 has been shown to be involved in many virus functions, including adsorption to host cells, virus virulence and interaction with several host proteins. Using a yeast two-hybrid screen, we have previously shown that the CSFV E2 specifically interacts with swine host protein medium-chain-specific acyl-Coenzyme A dehydrogenase (ACADM), an enzyme that catalyzes the initial step of the mitochondrial fatty acid beta-oxidation pathway. Here, we show that interaction between ACADM and E2 also happens in swine cells infected with CSFV using two different procedures: coimmunoprecipitation and a proximity ligation assay (PLA). In addition, the amino acid residues in E2 critically mediating the interaction with ACADM, M49 and P130 were identified via a reverse yeast two-hybrid screen using an expression library composed of randomly mutated versions of E2. A recombinant CSFV, E2ΔACADMv, harboring substitutions at residues M49I and P130Q in E2, was developed via reverse genomics from the highly virulent Brescia isolate. E2ΔACADMv was shown to have the same kinetics growth in swine primary macrophages and SK6 cell cultures as the parental Brescia strain. Similarly, E2ΔACADMv demonstrated a similar level of virulence when inoculated to domestic pigs as the parental Brescia. Animals intranasally inoculated with 10^5^ TCID_50_ developed a lethal form of clinical disease with virological and hematological kinetics changes undistinguishable from those produced by the parental strain. Therefore, interaction between CSFV E2 and host ACADM is not critically involved in the processes of virus replication and disease production.

## 1. Introduction

Classical swine fever virus (CSFV) is the etiological agent of a highly contagious disease of swine that can cause significant economic losses. CSFV is a small, enveloped positive-stranded RNA virus with a genome of approximately 12.5 kb. The virus genome is transcribed as a single open reading frame that encodes for a 3898-amino-acid polyprotein that, after its cleavage, produces 4 structural and 7/8 nonstructural proteins [1]. The four structural proteins are the core protein, associated with the viral RNA, and the three glycoproteins (Erns, E1 and E2) are associated with the virus envelope. In the last few years, several studies have focused on the role of these proteins in processes such as virus replication and virus virulence in the natural host, the domestic pig [2,3,4,5,6,7,8,9,10]. The analysis of the interaction between CSFV and host proteins and the potential role of those interactions in virus function have received attention in recent years. Several host proteins specifically interacting with structural CSFV proteins have been shown. As examples, SUMO1 (small ubiquitin-related modifier 1), IQGAP1 (IQ motif-containing GTPase-activating protein 1), UBC9 (Ubiquitin-Conjugating enzyme 9) and HB (Hemoglobin subunit beta) proteins [11,12,13,14] have been shown to interact with CSFV core protein. The structural protein E^rns^ has been demonstrated to interact with the Laminin receptor protein [15]. In addition, CSFV p7 has been shown to specifically interact with the calcium-signal-modulating cyclosphilin ligand (CALMG), the microtubule-associated protein RP/EB family member 1 (MAPRE1) and the voltage-dependent anion channel 1 (VDAC1) [16,17]. Additionally, E2 has also been demonstrated to interact with several host proteins, such as cellular actin [18], Anx2 (Annexin 2) [19], Trx2 (Thioredoxin) [20], MEK2 (mitogen-activated protein kinase 2) [21], PPP1CB (protein phosphatase 1 catalytic subunit beta) [22], SERTAD1 (SERTA-domain-containing protein 1) [23], CCDC115 (coiled-coil domain-containing 115) [24] and DCTN6 (dynactin subunit 6) [25]. Importantly, many of these interactions have been shown to have a critical role in the virus replication cycle or in virus virulence in swine.

Using a yeast two-hybrid approach, we previously reported that CSFV E2 specifically interacts with the swine host protein medium-chain-specific acyl-Coenzyme A dehydrogenase (ACADM), an enzyme that catalyzes the initial step of the mitochondrial fatty acid beta-oxidation pathway [26]. Here, we confirm that the interaction between CSFV E2 and ACADM, initially identified in a yeast two-hybrid model, occurs in cells infected with CSFV. Two independent methodologies, coimmunoprecipitation and a proximity ligation assay (PLA), were successfully used to detect E2-ACADM interaction in a cell line of swine origin, SK6. Furthermore, the amino residue in E2 critical to mediating the interaction with ACADM was identified, and based on that information, a recombinant virus harboring substitutions in those two E2 residues was developed via reverse genomics using as a template the highly virulent Brescia isolate. This recombinant mutant CSFV showed similar kinetics growth in swine primary macrophages and SK6 cell cultures and demonstrated an equal level of virulence when inoculated to domestic pigs to the parental Brescia strain. Therefore, interaction between CSFV E2 and host ACADM is not critically involved in the processes of virus replication and disease production.

## 2. Materials and Methods

### 2.1. Viruses and Cells

SK6 cell line cultures [4] were used to develop the recombinant virus, ensure stock virus production and perform virus titrations. SK6 cells were maintained in Dulbecco’s Minimal Essential Media (DMEM) (Gibco, Grand Island, NY, USA) containing 10% of BVDV free fetal calf serum (FCS) (Atlas Biologicals, Fort Collins, CO, USA). The parental virus is derived from an infectious clone encoding the CSFV Brescia strain (BICv) [2]. Prescence of viral infection was detected after 4 days in culture using the E2-specific CSFV monoclonal antibody WH303 [2] and the immunoproxidase Vectastain ABC Kit (Vector Laboratories, Burlingame, CA, USA). Virus titers were calculated and expressed as previously described [27] with a sensitivity of ≥1.8 tissue culture infectious doses (TCID)_50_/mL.

### 2.2. Coimmunoprecipitation

Interaction between host ACADM and CSFV E2 detected via coimmunoprecipitation using the Pierce Co-immunoprecipitation Kit (Thermo Fisher Scientific, Waltham, MA, USA) as recommended by the manufacturer. Mock and infected cell extracts were washed with ice-cold PBS and then lysed using the Pierce lysis buffer with protease inhibitors (Roche, Basel, Switzerland). The anti-E2 WH303 [28] monoclonal antibody was first conjugated to the Pierce beads and then incubated with the cell lysates overnight. The beads were then washed with Pierce Lysis buffer with 0.1% Triton-X-100 (Sigma-Aldrich, St. Louis, MO, USA) and protease inhibitors (Roche, Basel, Switzerland) and finally eluted in Pierce elution buffer. Preparations were run on NuPAGE 4–12% *w*/*v* Bis-Tris gels (Invitrogen, Carlsbad, CA, USA) and then transferred to polyvinylidene difluoride (PVDF) membranes. Immunodetection of ACADM was performed using a polyclonal anti-ACADM reagent (cat# PA5-70624, Thermo Fisher Scientific, Waltham, MA, USA). The monoclonal antibody WH303 [28] was used for the detection of CSFV E2 protein, and the Pierce Goat Anti-Mouse and Anti-Rabbit IgG peroxidase conjugated was used as a secondary antibody (Cat #31430 and 31460, respectively, from Thermo Fisher Scientific, Waltham, MA, USA). Western blots were imaged using an Azure C400 and analyzed with cSeries capture software (Azure Biosystems, Dublin, CA, USA, Cat #31430 and 31460, respectively, from Thermo Fisher Scientific, Waltham, MA, USA).

### 2.3. Proximity Ligation Assay

The interaction between host ACADM and CSFV E2 was also assessed using the Proximity Ligation Assay (PLA) [24]. The PLA was performed in triplicate using the Duolink-PLA Kit (Sigma-Aldrich, St. Louis, MO, USA) as recommended by the manufactures. Briefly, SK6 cells were seeded on 12 mm round coverslips (Thomas Scientific, Swedesboro, NJ, USA) in 24-well plates (Corning, Corning, NY, USA) at a concentration of 25,000 cells/well and were infected at an MOI of 10 for 24 h. Infected cells (along with mock-infected control preparation) were then fixed with 4% formaldehyde w/v in PBS for 20 min at room temperature. Cells were then permeabilized using PBS with 0.3% Triton-X-100 for 10 min and treated with Duolink blocking buffer for 30 min at 37 °C. Cell preparations were then incubated with the corresponding primary antibodies, anti-E2 WH303 [28] and Anti-ACADM (Abcam cat# EPR3708), at 4 °C for 1 h, washed twice with Duolink (Sigma-Aldrich, St. Louis, MO, USA) wash buffer A and incubated with the PLUS and MINUS PLA probes for 1 h at 37 °C. Reactions were then washed twice with Duolink Wash Buffer A, followed by an incubation at 37 °C with Duolink ligase in ligation buffer for 30 min, washed twice with Duolink Wash Buffer A and then incubated with Duolink polymerase in amplification buffer at 37 °C for 100 min. Fixed cells were then washed twice with Duolink Wash Buffer B and mounted with Duolink PLA mounting medium with DAPI.

### 2.4. Yeast Two-Hybrid Screening for Disruption of the E2-ACADM Reactivity

Plasmids harboring the E2 gene fused to the Gal4-binding domain (E2-BD) and the ACADM gene fused to the Gal4 activation domain (ACADM-AD) were synthesized (Epoch Life Sciences, Sugar Land, TX, USA). The plasmids used in this study were as follows. The T-antigen protein fused to the Gal4 activation domain (PGADT7). Human Lamin C (Lam-BD), a common negative control in the yeast two-hybrid system, fused to the Gal4-binding domain. Hypoxanthine-guanine phosphoribosyltransferase fused to the Gal4 activation domain (HPRT1-AD) as a positive control in terms of interacting with CSFV E2, as previously described [22,25]. E2-BD was randomly mutated as previously described [29]. The random E2 mutant library was then co-transformed into yeast strain AH109 along with ACADM with a transformation rate of at least 1 × 10^6^. Individual colonies were screened as previously reported [24], where plasmids were recovered and sequenced as previously described [25]. In recovered plasmids where only individual amino acids were mutated, the E2 mutant plasmids were re-tested individually via co-transformation with ACADM, HPRT1-AD (hypoxanthine phosphoribosyl transferase 1), as previously identified and described [26].

### 2.5. Construction of CSFV E2ΔACADM Mutant

An infectious clone (IC) harboring the complete genome of the virulent CSFV Brescia isolate (pBIC) [2] was used to include amino acid substitutions in the genomic area encoding for structural glycoprotein E2 to disrupt the E2-ACADM interaction. Substituted residues were identified by the reverse yeast two-hybrid approach [24]. Substitutions of E2 residue M49I and P130Q were incorporated into the native E2 amino acid sequence to obtain the E2ΔACADM construct. The E2ΔACADM plasmid was obtained via DNA synthesis (Epoch Life Sciences, Sugar Land, TX, USA).

The IC containing the E2ΔACADM construct was transfected into SK6 cells in 6-well plates and incubated for 4 days at 37 °C and 5% CO_2_. Expression of the E2 protein was detected via immunoperoxidase staining using E2-specific monoclonal antibody WH303, as described above. Recombinant virus E2ΔACADM was then harvested, and stocks were kept at −70 °C until use.

### 2.6. Animal Infection

The assessment of the virulent phenotype of the recombinant E2ΔACADM virus was performed using 30–40 lbs commercial breed swine. Recombinant E2ΔACADM or the parental BIC virus (BICv) was inoculated intranasally (IN) at a dose of 10^5^ TCID_50_. Animals were observed daily until the end of the experiment to detect the appearance of clinical signs associated with the disease (anorexia, depression, fever, purple skin discoloration, staggering gait, diarrhea and cough) and body temperature. Blood samples were obtained from the anterior vena cava in EDTA-containing Vacutainer tubes at the post-challenge time point, as described in the corresponding figures. Hematological cell profiles were obtained using a Beckman Coulter ACT (Beckman, Coulter, CA, USA). Experiments were performed under biosafety level 3 conditions in the animal facilities at Plum Island Animal Disease Center, following protocols approved by the Institutional Animal Care and Use Committee (number 171.12-21-R, approved on 12 September 2021).

## 3. Results and Discussion

### 3.1. CSFV E2 and ACADM Interact in CSFV-Infected Cells

**Figure 1:**
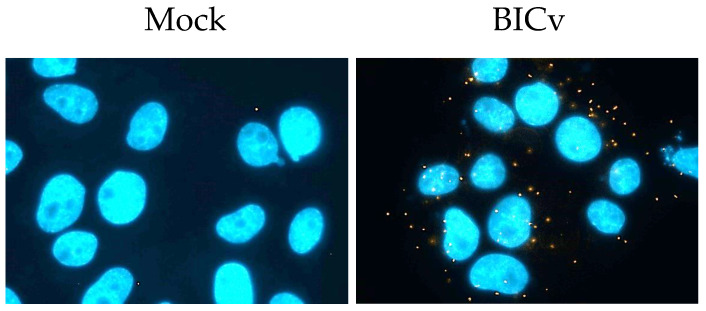
E2 and ACADM interaction were tested using a proximity ligation assay (PLA) in CSFV-infected SK6 cells. This interaction was determined using a PLA in SK6 cells that were either mock-infected or infected with CSFV for 24 h using CSFV BICv (MOI = 10). Magnification, ×1000. The figure shows representative results from one experiment, which was performed three times, with at least 100 cells analyzed in each experiment.

The interaction between CSFV E2 and ACADM was previously described using the yeast two-hybrid system [26]; therefore, it was necessary to extend these results and assess if the interaction between these two proteins actually takes place in cells infected with CSFV. To confirm the yeast two-hybrid results, two different approaches were used to determine if this interaction also occurs during CSFV cell infection: a proximity ligation assay (PLA) [30] and coimmunoprecipitation. For the PLA, which allows the identification of transient protein–protein interactions, SK6 cells were infected at a multiplicity of infection (MOI) of 10 with BICv. Samples were harvested at 24 h post-infection (hpi) and processed as described in the Materials and Methods section. The results from the PLA assay show that E2 and ACADM interacted in SK6 cell cultures infected with CSFV and that the interaction occurs as distinct punctate spots in the cytoplasm of infected cells, which was not observed in mock-infected cell preparations (Figure 1). This result demonstrates that E2 specifically interacts with ACADM, confirming that the interaction observed in the yeast two-hybrid system also occurs in CSFV-infected swine cells.

**Figure 2:**
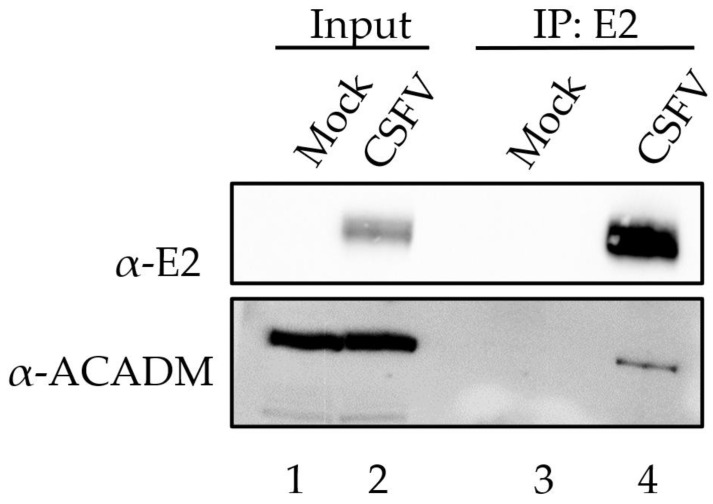
Coimmunoprecipitation was performed using E2 antibodies, and the presence of ACADM was tested for in CSFV-infected or mock-infected SK6 cells. The input cell lysate was compared with the IP. Protein extracts from both input and the IP were blotted for ACADM, as indicated by bands observed at the expected molecular weight of 45 kDa.

To further confirm the results obtained using the yeast two-hybrid screen and PLA, we performed coimmunoprecipitation (CoIP) studies between ACADM and E2 in extracts of SK6 cells infected with CSFV. The CoIP experiments were performed using antibodies specific for ACADM and E2. SK6 cells were infected with the CSFV strain BICv at an MOI of 10. Samples were then harvested after 24 h and CoIP was performed as described in the Materials and Methods section. Cell lysates from both the mock-infected and infected cell cultures were harvested, immunoprecipitated with the anti-E2 monoclonal antibody WH303 and then subjected to Western blotting with an ACADM-specific antibody. A single band showing a molecular mass of approximately 45 kDa (which corresponds to the predicted molecular weight calculated from its amino acid sequence) of ACADM was clearly identified (Figure 2). These results demonstrate that in CSFV-infected cell cultures, the structural protein E2 can be coimmunoprecipitated along with host ACADM, confirming the discovery from the yeast two-hybrid system. Therefore, using two different and independent methodologies (PLA and CoIP) it is shown that the E2 and ACADM proteins interact in CSFV-infected cells.

### 3.2. CSFV E2 Residues Critical for ACADM Interaction

The use of recombinant viruses containing amino acid substitutions to disrupt the interactions between a virus protein and its host cell ligand is valuable to examine the potential role of specific E2-host ligand interactions in a number of virus functions [22,23,24,25,29]. We noted earlier that the interaction between E2 and ACADM appears to be conformation-dependent since alanine scan mutagenesis failed to map E2 residues critical for mediating the interaction [26]. We used alanine scan mutagenesis to successfully map amino acid residues in virus proteins shown to interact with host protein ligands [11,24,25]. Then, the amino acid residues of CSFV E2 involved in the interaction with host ACADM were identified using the reverse yeast two-hybrid methodology as it was described in a previous study [25]. This methodology assesses the ability of ACADM to react with a collection of randomly mutated versions of E2 harboring approximately five amino acids that are randomly mutated. The mutated forms of E2 lacking interaction with ACADM were also evaluated for their ability to react with protein HPRT1, which is another host protein specifically recognizing E2, to eliminate the possibility that the random residue substitutions in E2 may result in gross conformational changes in the protein, disrupting the overall structure of E2 and resulting in a nonspecific loss of protein binding (Table 1). Under these experimental conditions, only one mutated form of E2 fulfilled all of the criteria while still losing the ability to bind ACADM1. This mutated version of E2, which possesses residue substitutions at positions M49I and P130Q, was further used to analyze the role of the ACADM–E2 interaction in the processes of CSFV replication and virulence.

### 3.3. Replication of the E2ΔACADM Mutant in Cell Cultures

**Figure 3:**
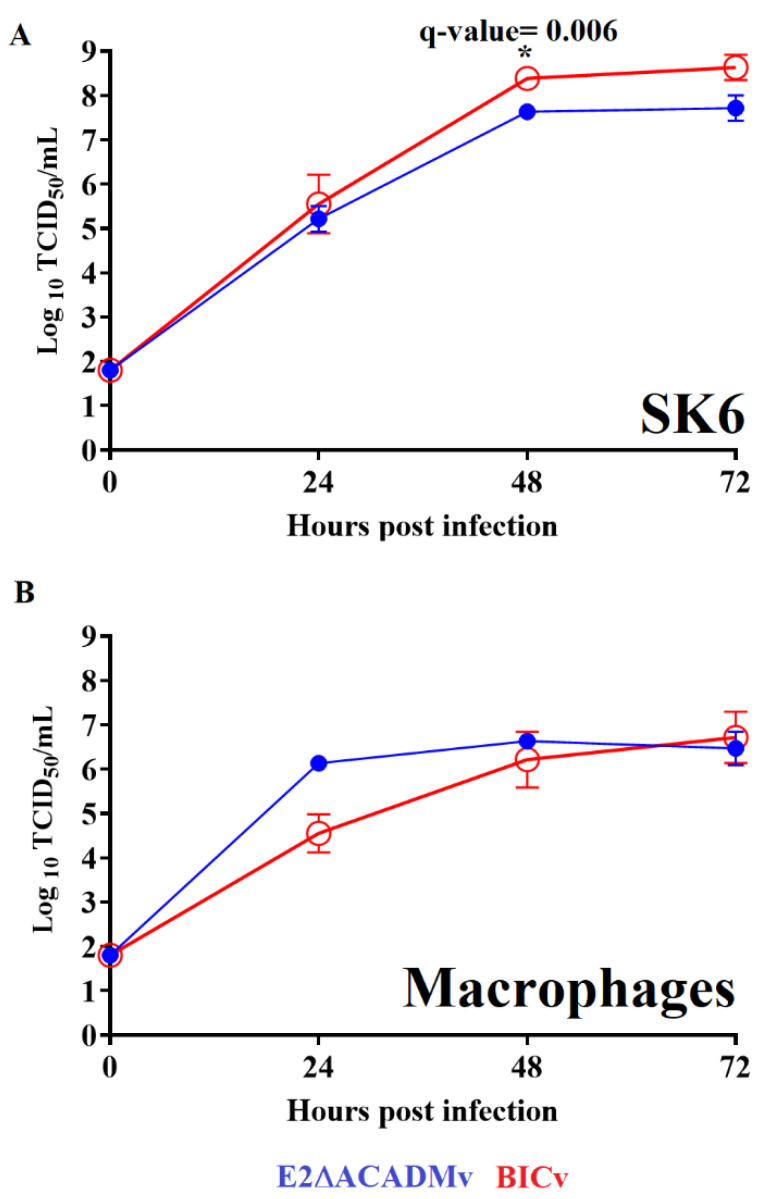
In vitro growth kinetics were determined using (**A**) SK6 cells and (**B**) primary cultures of swine macrophages for E2ΔACADMv and parental BICv (MOI = 0.01). Samples were obtained from two independent experiments performed in triplicate at the time points indicated and titrated in the corresponding cell substrate. The sensitivity obtained using this methodology for detecting the virus was ≥log10 1.8 HAD_50_/mL. An unpaired *t* test using the two-stage step-up method (Benjamini, Krieger and Yekutieli) was conducted to assess statistical differences in viral yields between E2ΔACADMv and BICv at different time points in both SK6 cells and primary porcine macrophages. The significance of this observation was evaluated using the false discovery rate method (FDR), with *q*-values < 0.05 (*) considered significant.

Based on the information obtained from the reverse yeast two-hybrid methodology, a recombinant CSFV harboring a mutated version of E2, which possesses residue substitutions at positions M49I and P130Q, was developed (E2ΔACADMv) via reverse genetics. The growth characteristics of the mutant virus E2ΔACADMv were compared with those of parental BICv (the pBIC-derived virus) in a multiple-step growth curve using both SK6 and primary swine macrophage cultures (Figure 3). Cell cultures of both type of cells were infected at an MOI of 0.01 TCID_50_, and samples were collected daily until 72 h post-infection (hpi). BICv displayed a slightly more efficient replication in SK6 cells, reaching virus yields that were almost ten times higher than those of E2ΔACADMv. Conversely, when the grow ability of BICv and E2ΔACADMv were compared in primary macrophage cultures, similar growth kinetics were observed for both viruses. Therefore, introduction of mutations in E2 that disrupt its interaction with host ACADM in the yeast two-hybrid system appears to slightly affect virus replication in SK6 cells but not in primary cultures of swine macrophages. Regardless of the statistical differences observed at some of the time points at which SK6 cells were tested, the results do not appear to support differences in biological significance in terms of virus yield between the mutant and the parental virus.

### 3.4. Assessment of E2∆ACADM Virulence in Swine

**Figure 4:**
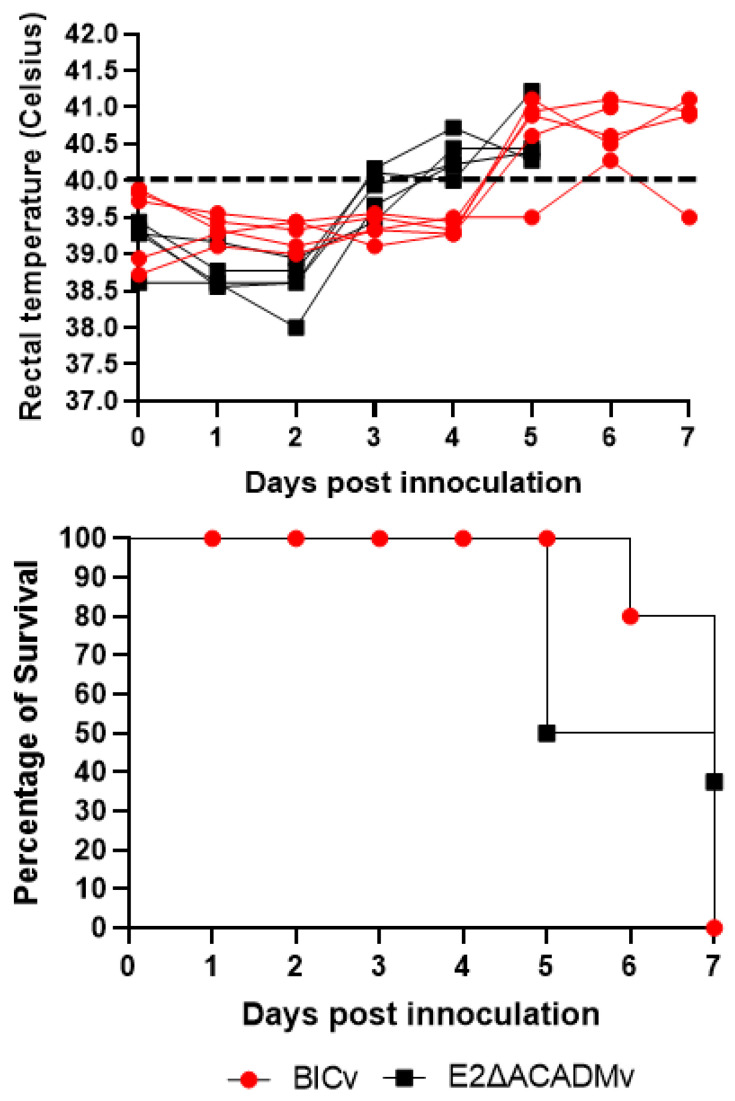
Evolution of body temperature and lethality in animals (5 animals/group) IN infected with 10^5^ HAD_50_ of either E2ΔACADMv or parental BICv. After analyzing the results using two methods, the long-rank (Mantel–Cox) and the Gehan–Breslow–Wilcoxon tests, there were no observed significant statistical differences between the different groups in terms of temperatures or lethality.

To assess the possible role of the amino acid substitutions introduced in glycoprotein E2 of E2ΔACADMv during virus replication as well as in the production of disease in the natural host, E2ΔACADMv was used to infect domestic pigs under experimental conditions. Two groups, composed of five animals weighing 30–40 lbs each, were intranasally (IN) infected with 10^5^ TCID_50_ of either E2ΔACADMv or the parental BICv, and the appearance of clinical signs associated with CSF was monitored on a daily basis. All the animals infected with the virulent BICv had disease onset around day 5 pi with a quick evolution of the disease, showing an increase in body temperature to over 40 °C, with all animals being euthanized by days 6–7 pi (Figure 4).

The animals infected with E2ΔACADMv also showed a lethal form of the disease, showing the appearance of the clinical signs of the disease slightly earlier than animals inoculated with the parental virus. Onset of the disease was detected between days 3 and 4 pi, after which the disease evolved to severe forms, and all animals were euthanized on days 5–7 pi. Therefore, amino acid substitutions introduced in the glycoprotein E2 of E2ΔACADMv did not alter its virulence in domestic pigs.

**Figure 5:**
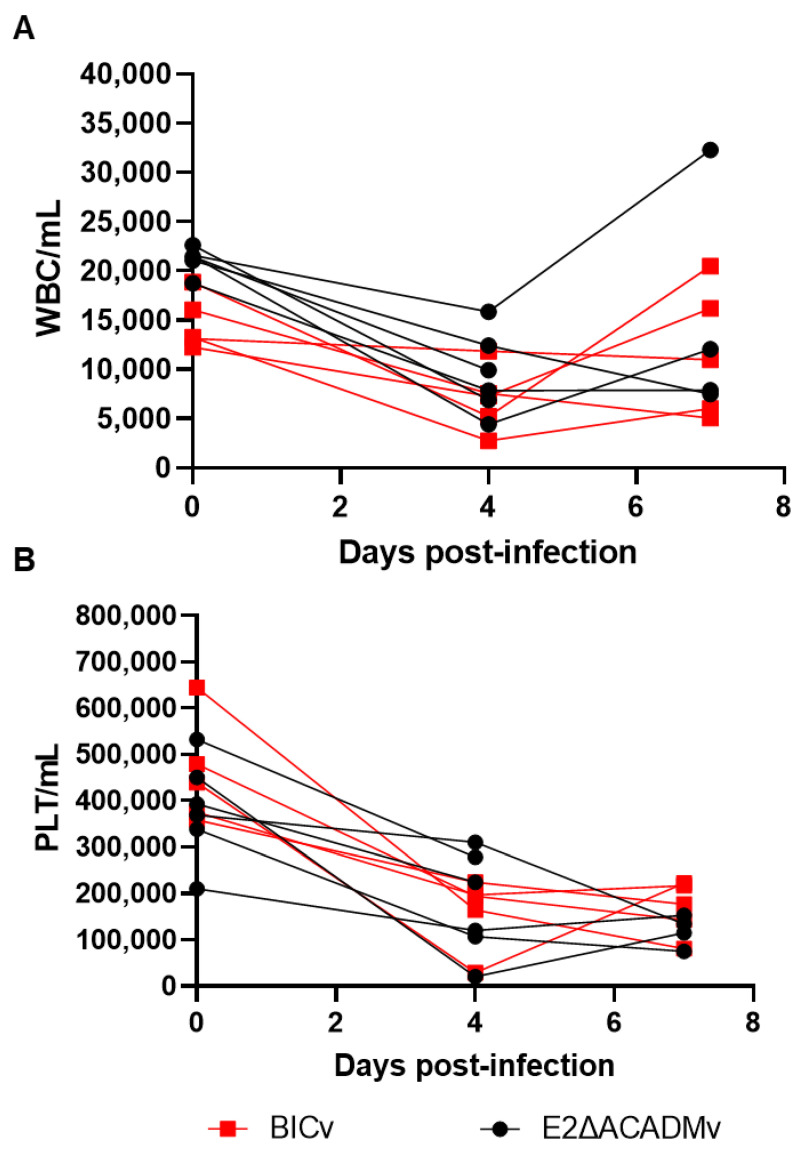
Hematological changes in pigs IN inoculated with 10^5^ TCID_50_ of either E2ΔACADMv or BICv. Each curve represents individual animal values expressing concentration of (**A**) white blood cells (WBCs) and (**B**) platelets (PLTs) /mL of blood.

Hematological values changed over the evolution of the clinical disease. White blood cell and platelet counts had drastically dropped by day 4 pi in animals inoculated with either of the viruses, with no significant changes seen until death (Figure 5).

**Figure 6:**
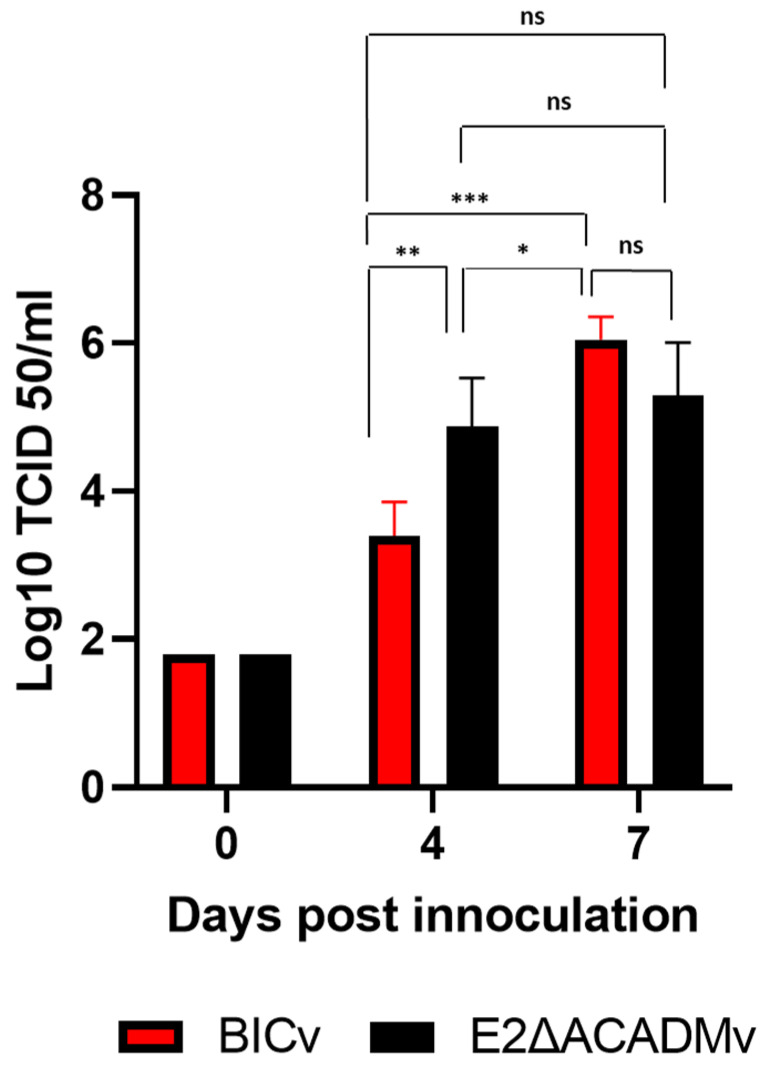
Viremia titers detected in pigs IN inoculated with 10^5^ TCID_50_ of either E2ΔACADMv or BICv. Each bar represents the average and standard deviation of animals in each group at the corresponding time point. * *p* ≤ 0.05, ** *p* ≤ 0.01, *** *p* ≤ 0.001, ns, not significant.

The capacity of recombinant E2ΔACADMv to replicate during the experimental infection in pigs was evaluated by comparing the viremia titers to those detected in the animals inoculated with BICv (Figure 6). Animals infected with the virulent parental BICv presented viremia titers ranging from 10^2.8-^ to 10^3.8^ TCID_50_/mL up to day 4 pi. Viremia values increased from 10^5.8^ to 10^6.55^ TCID_50_/mL until the day they were euthanized. The animals inoculated with E2ΔACADMv presented higher viremia titers up to day 4 pi, ranging from 10^4.8^ to 10^5.8^ TCID_50_/mL, and remained at those levels until being euthanized due to the severity of the clinical signs. Therefore, E2ΔACADMv does not present a significantly different virulent phenotype from that of the parental virulent BICv.

The role of the interaction between virus and host proteins in critical processes such as virus replication and production of disease in domestic pigs infected with CSFV is still not well understood. Although the number of studies on the identification and characterization of those interaction has increased in last years, the importance of those interactions regarding the molecular pathways utilized by the virus for replication and/or to manipulate the host immune response for the virus’s own survival is not clear. In prior studies, we reported the specific interaction of several host proteins with CSFV proteins. For instance, we identified and characterized the interaction of the structural CSFV core protein with host proteins SUMO1, IQGAP1, UBC9 and OS9 [11,12,13]. In addition, we demonstrated the interaction of nonstructural viroporin p7 with host CAMLG [16] as well as the interaction of the major structural glycoprotein E2 with host proteins PPP1CB [22], DCTN6 [25], SERTAD1 [23] and CCDC115 [24]. These interactions between virus and host proteins usually affect virus replication and, importantly, in some of these cases, may have an important role in the production of disease in pigs [11,12,13,25]. In addition, other laboratories have demonstrated that structural protein E2 interacts with several host proteins, such as cellular actin [18], Anx2 [19], Trx2 [20] and MEK2 [21]. Therefore, identifying and understanding the involvement of these virus–host interactions is critical to enhance the understanding of the molecular mechanisms involved in viral infection of the natural host.

Here, we reported the identification and characterization of the interaction between the CSFV E2 protein and host protein ACADM, an enzyme that catalyzes the initial step of the mitochondrial fatty acid beta-oxidation pathway.

The results reported here are the first indication that the cellular protein ACADM is involved in CSFV during cellular infection and interacts with viral protein E2. Results demonstrate that mutations introduced in the structural protein E2 do not provoke a significant change in the CSFV phenotype in terms of affecting virus replication in vitro or virus virulence in vivo. It should be mentioned that amino acid mutations leading to disruption of the E2-ACADM interaction in the yeast two-hybrid system may not necessarily produce the same effect during E2ΔACADMv infection, and further experiments should be performed in order to confirm this. Gaining knowledge on the role of specific amino acid residues of CSFV proteins in important processes such as virus replication or virulence is necessary to develop potential novel methods to block or decrease virus infection in the natural host. A better understanding of the host factors interacting with virus proteins at the amino acid level is a significant step towards a better understanding of the impact of potential mutations that occur between different virus strains that continue to emerge from CSFV, often exhibiting different degrees of viral virulence and pathogenesis for unknown reasons. However, as we explore the roles of individual residues in the CSFV genome, in the future it may be possible to predict the pathogenesis and characteristics of emerging strains of CSFV before the occurrence of outbreaks.

## Figures and Tables

**Table 1:** The yeast two-hybrid system was used to confirm protein–protein interaction between CSFV native protein E2 or mutant E2-∆ACADM (M49I and P130Q) with Lam coupled to the Gal4-binding domain, ACADM or HPRT coupled to the Gal4 activation domain. Results for yeast growth on selective media SD-Ade/His/Leu/Trp.

			Gal4 Binding Domain	
		Lam	E2	DACADM
**Gal4**	T-ag	-	-	-
**Activation**	ACADM	-	+	-
**Domain**	HPRT	-	+	+

## Data Availability

All data has been provided in the article.
